# Use of liquid biopsies to monitor disease progression in a sarcoma patient: a case report

**DOI:** 10.1186/s12885-016-2992-8

**Published:** 2017-01-06

**Authors:** Heidi M. Namløs, Olga Zaikova, Bodil Bjerkehagen, Daniel Vodák, Eivind Hovig, Ola Myklebost, Kjetil Boye, Leonardo A. Meza-Zepeda

**Affiliations:** 1 Department of Tumor Biology, Institute for Cancer Research, The Norwegian Radium Hospital, Oslo University Hospital, Oslo, Norway; 2 Department of Surgery, Oslo University Hospital, Oslo, Norway; 3 Department of Pathology, Oslo University Hospital, Oslo, Norway; 4 Institute of Cancer Genetics and Informatics, The Norwegian Radium Hospital, Oslo University Hospital, Oslo, Norway; 5 Norwegian Cancer Genomics Consortium (CancerGenomics.no), Oslo, Norway; 6 Department of Oncology, Oslo University Hospital, Oslo, Norway; 7 Genomics Core Facility, Department of Core Facilities, Institute for Cancer Research, The Norwegian Radium Hospital, Oslo University Hospital, Oslo, Norway

**Keywords:** Circulating cell-free DNA, ctDNA, ThunderBolts, *KRAS*, *NF1*, Cancer, Case report

## Abstract

**Background:**

Many patients experience local recurrence or metastases after receiving potentially curative treatment, and early detection of these events is important for disease control. Recent technological advances make it possible to use blood plasma containing circulating cell-free tumour DNA (ctDNA) as a liquid biopsy. In this case report we show how serial liquid biopsies can be used to monitor the disease course and detect disease recurrence in a sarcoma patient.

**Case presentation:**

A 55-year-old male presented with a rapidly growing, painful palpable mass in the left groin region, and a biopsy revealed a high-grade malignant spindle cell sarcoma. No metastases were detected on radiologic imaging scans. Using targeted resequencing with a custom 900 cancer gene panel, eight somatic mutations among them *KRAS* and *NF1*, were identified in the primary tumour. Targeted resequencing of plasma cell-free DNA (ctDNA) collected before and after surgery and at disease progression confirmed the presence of six of eight mutations at all three time points. The ctDNA level, estimated from the somatic allele frequencies of these six mutations, was high in plasma taken at the time of surgery, at levels similar to the primary tumour. Detection of low levels of ctDNA three days after surgery indicated persistent microscopic disease. Repeated radiologic imaging six weeks postoperatively showed widespread metastatic disease in the lungs, skeleton and the pelvic region. At this time point there was a dramatic increase in the ctDNA level, reflecting the disease progression of the patient. The patient had an unusually aggressive cancer, and succumbed to the disease 13 weeks after surgery.

**Conclusions:**

This case report demonstrated that targeted resequencing of ctDNA from longitudinal collected plasma can be used to monitor disease progression in a soft tissue sarcoma patient, including manifestation of metastatic disease. The ctDNA represented the genomic profile of the tumour, supporting clinical use of liquid biopsies to identify tumour-specific mutations as well as recurrent disease.

**Electronic supplementary material:**

The online version of this article (doi:10.1186/s12885-016-2992-8) contains supplementary material, which is available to authorized users.

## Background

Cancers arise through a sequential alteration of the genome, resulting in a heterogeneous tumour that continuously evolves as the disease progress. Material for diagnostic examination of solid tumours is routinely obtained through fine-needle biopsies. This procedure gives limited amount of material, and only provides a single snap-shot of the genetic alterations in a restricted part of the tumour. After treatment with curative intent, radiological imaging of patients to monitor local or distant recurrences is not done on a routine basis, and imaging methods also have limited sensitivity to detect micrometastases.

Recent advances make it possible to use blood plasma as a liquid biopsy, examining the circulating cell-free DNA (cfDNA) shed from both normal and tumour cells into peripheral blood [1–3]. The circulating cell-free tumour DNA (ctDNA) has been shown to contain the various tumour-specific alterations seen in the primary and metastatic tumours, and may more accurately represent the genetic profile of the whole tumour mass compared to DNA from a single biopsy of a heterogeneous lesion [4]. By repeated sampling of liquid biopsies, somatic mutations identified in cfDNA can be used as unique non-invasive tumour-specific biomarkers for monitoring tumour burden throughout the disease course. Similar procedures are now in use for screening of foetal genetic aberrations using the mothers blood, and in several cases aberrations from malignant tumours have been detected presymtomatic in pregnant women [5].

Several reports have demonstrated that high-throughput sequencing of cfDNA may be used for prognosis and molecular stratification, early detection of recurrence and metastasis, monitoring response to treatment and identification of resistance mechanisms [6]. Sequencing of cfDNA has been performed for cancers like colorectal, ovarian and breast, showing that the level of tumour-specific mutations reflects the course of the disease and the treatment response [7–9].

Sarcomas make up a heterogeneous group of malignant tumours of mainly mesenchymal origin. The overall five-year survival of all soft tissue sarcoma patients is approximately 70% [10, 11], and about 75% of soft tissue sarcomas are highly malignant. Soft tissue sarcomas often recur locally and/or metastasize, and the median time to local recurrence is around 1-1½ year and to metastasis about 1 year [12, 13], both decreasing long-term survival. From a molecular genetics perspective, sarcomas are genetic diverse and may have numerous somatic mutations [14]. The use of high-throughput sequencing of cfDNAs longitudinally collected during disease progression, making simultaneous screening for multiple mutations during the disease course possible, has not yet been reported for sarcomas. However, a recent study described exome sequencing of a primary soft tissue sarcoma tumour and a single plasma sample collected at time of metastasis, and showed that new mutations had appeared in ctDNA after progression on chemotherapy and targeted treatment [15]. Monitoring of disease burden in one osteosarcoma patient has previously been demonstrated using PCR to detect somatic rearrangements in plasma [16], and allele-specific PCR has been used to obtain mutation profiles from plasma of GIST patients [17].

As part of an ongoing prospective study (CircSarc), we collected primary tumour and plasma samples taken before and after surgery and at disease progression from a soft tissue sarcoma patient. Targeted resequencing was used to identify somatic mutations in the primary tumour and monitor the level of ctDNA from plasma samples during the course of the disease.

## Case presentation

**Fig. 1:**
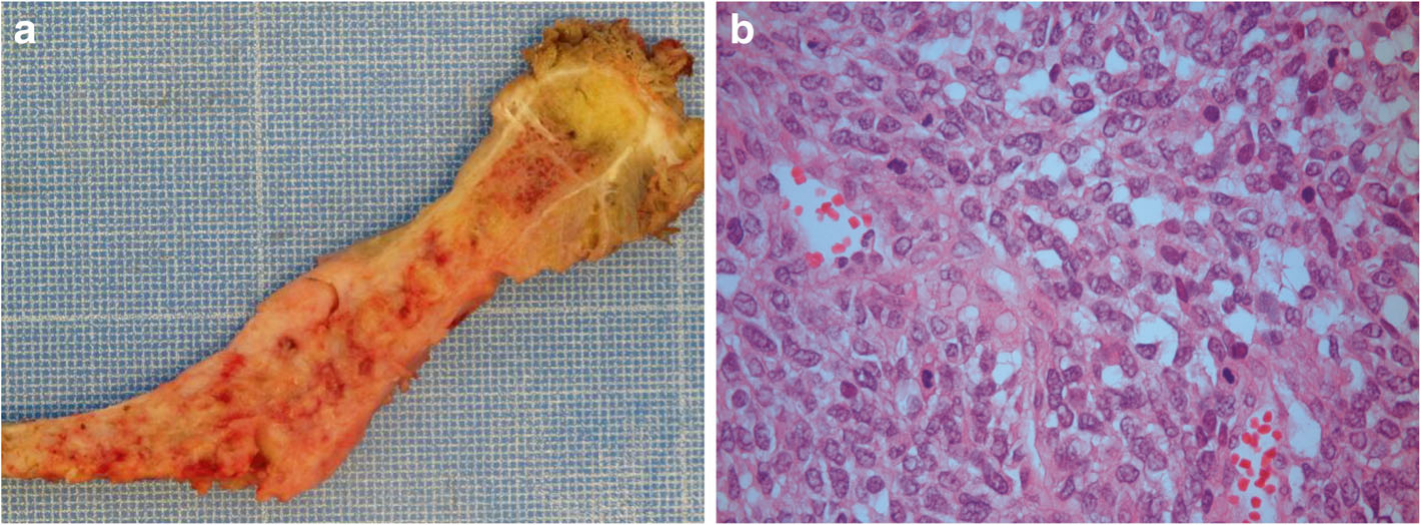
Examination of tumour tissue (**a**). Macroscopic examination of tumour in femur after hemipelvectomy. **b** Histological picture of heamatoxylin and eosin stained slide of patient biopsy material classified as undifferentiated spindle cell sarcoma

A 55-year-old male presented with a rapidly growing, painful palpable mass in the left groin region involving the femoral nerve and blood vessels. Magnetic resonance imaging performed 18 days before surgery revealed a 10.5 x 7.6 x 11.0 cm large intramuscular tumour. No metastases were detected on CT scans of the chest, abdomen and pelvic area performed 14 days before surgery. Microscopic evaluation of a biopsy revealed a high-grade malignant spindle cell sarcoma (Fig. 1). Due to extensive locoregional growth into the skeleton and intractable pain, a hemipelvectomy was performed. Small focus with metastatic disease was detected in two lymph nodes removed during the surgery. Macroscopic examination showed a well demarcated nodular tumour with white and fleshy cutting surface with small necrotic areas and bleeding. Immunohistochemical analysis showed positive finding for CD99 and AE1/AE3, and negative staining for S-100, SMA, EMA and CD31. Cytogenetic analysis showed massive clonal chromosomal rearrangements, and PCR and FISH were negative for fusion genes normally seen in synovial sarcoma. The differential diagnoses were synovial sarcoma and malignant peripheral nerve sheath tumour. Lymph node metastasis is more commonly seen in synovial sarcoma and the immunohistochemical finding is also in favour of a synovial sarcoma, but the genetic findings did not support that diagnosis. According to the WHO classification [18], the tumour was classified as an undifferentiated spindle cell sarcoma.

Targeted resequencing of the tumour and normal genomic DNA was performed following a SureSelect protocol (Agilent Technologies, Santa Clara, California, US) and a custom in-solution capture panel, containing exons of 900 cancer-related genes, developed by the Norwegian Cancer Genomics Consortium (NCGC, cancergenomics.no) [19]. The methods are provided in (Additional file 1). The sequencing revealed eight somatic mutations in the primary tumour. Among these, seven point mutations were identified in the genes *COL2A1* (intronic), *NF1* (p.K354R), *PTGS2* (intronic), *LRP2* (p.Q4132E), *KRAS* (p.G12V), *PRRC2C* (p.R1257G) and GATA6 (p.A29A), as well as a frameshift deletion in *PRG4* (p.R791fs) (Table 1 and Additional file 2). Copy number analysis revealed a homozygote deletion of *TP53* (Additional file 3). Targeted resequencing using a smaller ThunderBolts Cancer panel (Raindance Technologies, Billerica, Massachusetts, US) confirmed the identified *KRAS* mutation in the primary tumour at an allele frequency of 66%, similar to the 60% frequency found using the 900 gene panel (Additional file 2). The additional seven mutated genes were not included within the ThunderBolts Cancer panel.

**Table 1:** Overview of somatic mutations in primary tumour. The sequencing libraries were generated using the SureSelect^XT^ protocol and a 900 cancer-related custom SureSelect in-solution capture gene panel

Gene	Position	Reference allele	Mutated allele	Codon change	Allele frequency tumour	Depth tumour	Consequence
*COL2A1*	chr12:48372367	G	C	NA	96.0	725	Intron variant
*NF1*	chr17:29527612	A	G	p.K354R	92.9	438	Missense variant
*PTGS2*	chr1:186644059	C	A	NA	64.0	39	Intron variant
*LRP2*	chr2:170009376	G	C	p.Q4132E	61.0	443	Missense variant
*KRAS*	chr12:25398284	C	A	p.G12V	60.4	699	Missense variant
*PRRC2C*	chr1:171510380	A	G	p.R1257G	29.0	807	Missense variant
*GATA6*	chr18:19751192	G	T	p.A29A	18.9	920	Synonymous variant
*PRG4*	chr1:186277624	CGTACTACACCT	C	p.R791fs,p.R884fs,p.R832fs,p.R925fs	10.6	810	Frameshift variant & feature truncation

**Fig. 2:**
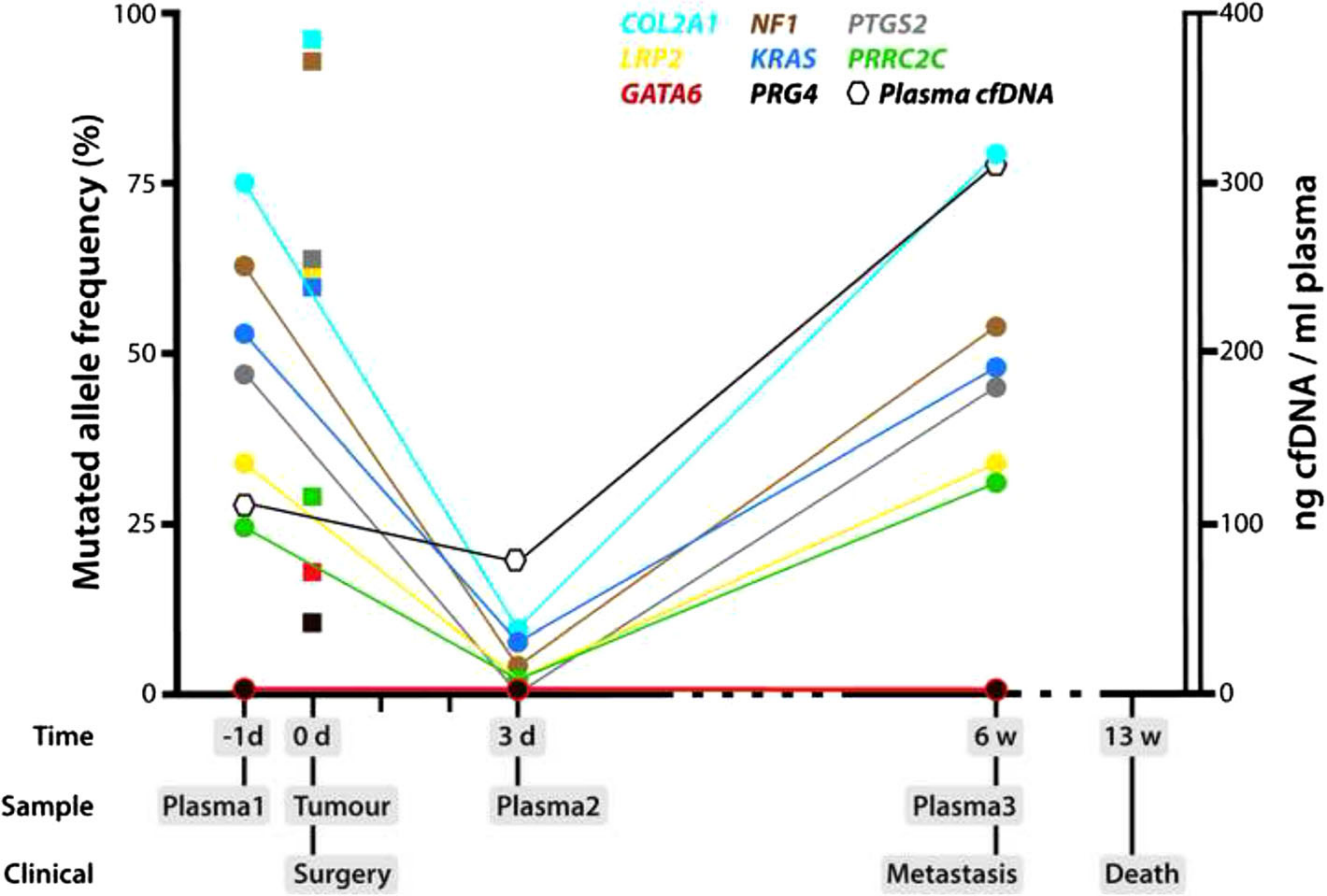
Levels of mutations in ctDNA and total cfDNA level in serial samples during disease progression. The allele frequency of mutations in *COL2A1*, *NF1*, *PTGS2*, *LRP2*, *KRAS* and *PRRC2C* was quantified in primary tumour (squares) at time of surgery (0d) and in serial plasma samples (circles) collected one day (−1d) before surgery, three days (3d) and six weeks (6w) after surgery using targeted resequencing. The total level of cfDNA (normal and tumour) was quantified in plasma samples (hexagon) using Qubit fluorometer. Distal metastases were identified after six weeks (6w), and the patient succumbed to the disease after 13 weeks (13w) with aggressive metastatic disease

The patient was scheduled for adjuvant chemotherapy, but repeated radiologic imaging six weeks postoperatively showed widespread macroscopic metastatic disease in the lungs and skeleton, as well as numerous soft tissue metastases in the pelvic region. Targeted resequencing of the plasma samples, using the NCGC 900 cancer gene panel, confirmed the presence of six of the eight above mutations in all three plasma samples with allele frequencies ranging from 2.1-75% (Fig. 2 and Additional file 2). The mutation levels in plasma before surgery were comparable to the ones in the primary tumour with a Pearson’s correlation of 0.93 between frequencies in tumour and plasma. The mutations in *GATA6* and *PRG4*, with the lowest allele frequencies in the primary tumour, were not detected in the plasma samples. The mean sequencing depth of the six detected mutations in all three plasma samples was 260x (11-474x), and the two undetected had a depth of 360x (199-433x). One new mutation in the splice site of *RAD52*, a C > T mutation with an allele frequency of 5.5%, was detected in the cfDNA collected before surgery. This region was sequenced at high coverage, but the mutation was not observed in the primary tumour nor in the plasma samples collected at later time points (Additional file 2).

Targeted resequencing using the ThunderBolts Cancer panel confirmed the presence of *KRAS* (p.G12V)) in all plasma samples (Additional file 2). No other mutations with >5% allele frequency were detected in more than one sample.

The level of total cfDNA was monitored during disease progression. High quantity of cfDNA was detected one day before surgery (110 ng/ml plasma), and a decrease was seen three days after surgery (76 ng/ml plasma). Six weeks after surgery, the quantity of cfDNA had increased to more than twice the initial level present before the surgery (316 ng/ml plasma) (Fig. 2).

The ctDNA level was estimated from the somatic allele frequency of the recurrent mutations in the genes *COL2A1*, *NF1*, *PTGS2*, *LRP2*, *KRAS* and *PRRC2C.* The ctDNA level in plasma collected one day before the surgery (Plasma1) was high, and comparable to the level in primary tumour. Three days after surgery, the ctDNA level had dropped, but was still detectable in plasma (Plasma2). In the sample collected six weeks after surgery (Plasma3), there was again an increase in ctDNA level similar to the levels before surgery (Fig. 2). When also taking into account the amount of cfDNA released, the number of mutated genomes per ml of plasma were three times higher at this time point than before surgery (Additional file 4). This reflected the disease progression of the patient and correlated with the tumour burden, as multiple distant metastases were detected at this time. The patient’s general condition was considered too poor for administering chemotherapy, and he succumbed to the disease 13 weeks after surgery.

## Discussions

In this study, we prospectively collected primary tumour and normal sample material at surgery and several plasma samples during the disease course of a high-grade soft tissue sarcoma patient. Targeted resequencing of the primary tumour and the normal sample identified eight somatic mutations of which six were also present in the plasma samples. Among the mutations, *KRAS* (p.G12V) and *NF1* (p.K354R) were predicted by dbNSFP [20] to have a deleterious effect on the protein function. It has been reported that simultaneous inactivation of *TP53* and activation of *KRAS* induced quick formation of spindle-cell sarcoma in soft tissues in double transgenic mice [21]. The homozygous deletion of *TP53* found in the primary tumour strengthens the histology observed in the primary tumour. The patient in our study had an unusually aggressive spindle-cell sarcoma, supporting *KRAS* not only as biomarker, but as a driving gene of the disease progression. *NF1*, a tumour suppressor that functions as a negative regulator of the Ras pathway, is among the most frequently mutated genes in several subtypes of sarcomas. Germline and somatic loss of *NF1* in neurofibromatosis patients cause malignant peripheral nerve sheath tumours [22] and GISTs [23]. In addition, somatic *NF1* mutations, including deletions, have been reported in a wide variety of paediatric and adult soft-tissue sarcomas with complex karyotypes [24, 25]. Although no therapeutics that target *KRAS* or *NF1* are available, our study shows that repeated sampling using liquid biopsies opens new possibilities to identify and monitor biomarkers that can be used in targeted therapies.

The synonymous mutation in *GATA6* and the frameshift deletion in *PRG4* could not be detected in the plasma samples. Although these regions had a high coverage, these mutations had the lowest allele frequency in the primary tumour and this may thus be a sensitivity issue. However, it is also possible that the cells containing these mutations did not release DNA into circulation. This shows that the ctDNA gives a good representation of the genomic profile of the tumour, but also emphasize the need to use several mutations when monitoring disease development. A mutation in *RAD52*, a gene involved in DNA recombination, was detected in the plasma collected before surgery, but was not observed in the primary tumour nor in the plasma sample collected after surgery or at recurrence. One explanation for this may be tumour heterogeneity where the cells containing this mutation were not present in the part of the primary tumour that was sequenced and was not retained during disease progression. Discrepancy of mutation pattern between DNA from primary tumours and plasma have also previously been reported [26, 27], showing that liquid biopsies may better capture all mutations present in the primary tumour and/or liver metastasis and can thus be used to overcome the challenges posed by intra-tumour genetic heterogeneity [4]. We show that ctDNA from plasma represents an attractive and easily available source of sarcoma tumour DNA, although it remains to be seen how ctDNA levels vary across patients and stages.

The allele frequencies of the six mutated genes in the cfDNA represent the ctDNA level during disease progression. Three days after surgery, ctDNA was still detectable in the liquid biospy. cfDNA has a rapid clearance, with reported half-life from 15 min [28] to 13 h for foetal cfDNA in plasma [29]. Although not detectable by CT before surgery, metastatic disease was detected in two lymph nodes removed during the hemipelvectomy, and a small amount of ctDNA detected was likely released from additional undiscovered local metastases that could not be detected by conventional diagnostic modalities. The patient had a very aggressive course of the disease, and metastases were detected both in soft tissue, skeleton and lungs only a few weeks after surgery. The plasma collected six weeks after surgery showed an increase in ctDNA relative to the levels before surgery, reflecting the presence of a tumour and rapid disease progression.

The cfDNA can originate from both normal and tumour cells. Based on the high mutated allele frequencies determined in plasma, the initial level of cfDNA is dominated by DNA from the tumour. Most of the cfDNA present three days after surgery is believed to originate from tissue injury and inflammation of normal cells as a consequence of the extensive surgery, which would explain the apparently higher normal contribution to the cfDNA at this time point. After six weeks, there was a large increase in cfDNA accompanied with an increase of the mutated allele frequencies. Thus, the quantities of cfDNA present in the plasma reflected the clinical status of the patient due to the fact that most of the cfDNA released during disease progression was tumour derived.

## Conclusions

This study is the first report of using targeted resequencing of cfDNA from serial plasma samples to monitor disease progression in a soft tissue sarcoma patient. The findings show that the level of tumour-specific mutations in liquid biopsies is correlated to disease course in sarcomas, including clinical manifestation of metastatic disease. The longitudinally collected ctDNA allow for near real-time monitoring of the tumour genome during disease progression, and the ctDNA gives a good representation of the genomic profile of the tumour supporting the use of ctDNA from plasma as a liquid biopsy.

## Additional files

Additional file 1 Somatic mutations identified in primary tumour and plasma samples using NCGC 900 and Thunderbolt Cancer Panel. (DOCX 18 kb)
Additional file 2 Somatic mutations identified in primary tumour and plasma samples using NCGC 900 and Thunderbolt Cancer Panel. (XLS 62 kb)
Additional file 3 Plot showing copy number of chromosome 17p, revealing homozygote deletion of the *TP53* gene. (TIF 1032 kb)
Additional file 4 Plot showing mutated genomes per ml of plasma, for the mutated genes, in serial plasma samples. (TIFF 283 kb)

## Abbreviations

BWA: Burrows-Wheeler alignment
cfDNA: Circulating cell-free DNA
*COL2A1*: Collagen, type II, alpha 1
ctDNA: Circulating cell-free tumour DNA
*GATA6*: GATA binding protein 6
GATK: The genome analysis toolkit
*KRAS*: Kirsten rat sarcoma viral oncogene homolog
*LRP2*: Low density lipoprotein receptor-related protein 2
NCGC: Norwegian cancer genomics consortium
*NF1*: Neurofibromin 1
*PRG4*: Proteoglycan 4
*PRRC2C*: Proline-rich coiled-coil 2C
*PTGS2*: Prostaglandin-endoperoxide synthase 2
*RAD52*: *RAD52* homolog (S. Cerevisiae)
*TP53*: Tumour protein P53
