# Supplementary material for: Use of liquid biopsies to monitor disease progression in a sarcoma patient: a case report
Source: BMC Cancer. 2017 Jan 6;17:29. doi: 10.1186/s12885-016-2992-8 (PMC5219677; doi:10.1186/s12885-016-2992-8)
Supplement: Additional file 1 — Somatic mutations identified in primary tumour and plasma samples using NCGC 900 and Thunderbolt Cancer Panel. (DOCX 18 kb) [file 12885_2016_2992_MOESM1_ESM.docx]

# **Material and methods**

### Patient material

Tumour material was fresh frozen at -80 ˚C immediately after surgery. Two tissue sections of the tumour were stained with haematoxylin and eosin, and the presence of representative tumour material was verified by the sarcoma pathologist (B.B.). EDTA blood samples were drawn from the patient one day before surgery (Plasma1), three days after surgery (Plasma2) and six weeks after surgery (Plasma3). All blood samples were processed within one hour.

### DNA isolation

DNA from fresh frozen tumour material consisting of >95 % tumour was isolated using the Wizard Genomic DNA Purification Kit (Promega, Fitchburg, Wisconsin, US) according to the manufacturer's protocol. The blood was centrifuged at room temperature at 820 x g for 10 min, and the plasma layer was re-centrifuged at room temperature at 16,000 x g for 10 minutes. The plasma was frozen at -80 ˚C, and after thawing the plasma was re-centrifuged at 16,000 x g for 5 minutes. cfDNA representing both normal and tumour DNA, was isolated from 2 ml plasma using QIAamp Circulating Nucleic Acid Kit (Qiagen, Hilden, Germany) and stored at -20 ˚C. The buffy coat layer from the first centrifugation at 820 x g, representing normal cells, was frozen at -80 ˚C. Genomic DNA was isolated using DNeasy Blood (Qiagen) according to the manufacturer's protocol. Genomic DNA and cfDNA were quantified using a Qubit fluorometer from Invitrogen (Thermo Fisher Scientific, Waltham, Massachusetts, US). The fragment length of the cfDNA was measured using Tapestation D1000 screen tape (Agilent Technologies, Santa Clara, California, US).

### Sequencing of primary tumour and normal sample

Sequencing libraries were prepared from 1 µg genomic DNA from the tumour and normal sample following the SureSelect^XT^ protocol (Agilent Technologies) and with 50 ng genomic DNA following the SureSelect^QXT^ protocol. A custom SureSelect (Agilent Technologies) in-solution capture panel (NCGC 900), developed by the Norwegian Cancer Genomics Consortium (NCGC, cancergenomics.no), was used to enrich for exons of 900 cancer-related genes, selected promoters and introns frequently involved in fusions. The libraries were sequenced paired-end (2 x 100 bp) on a HiSeq2500 (Illumina Inc., San Diego, California, US) using TruSeq SBS v3 chemistry. Real-time analysis and base calling were conducted by Illumina’s software packages HSC2.0.2/RTA1.17.21.3. Raw reads were processed using the Illumina CASAVA (v. 1.8.2) to demultiplex data and filter out the low-quality reads.

The reads were mapped with BWA mem[2] to the human reference genome (build b37 with an added decoy contig, obtained from[3]). Sorting and duplicate marking were performed on the initial alignments with Picard tools[4]. GATK tools[5] were used for two-step local realignment around indels, with tumour/normal matching samples. Pair-end read information was checked for inconsistencies with Picard and base-quality recalibration was performed by GATK. Somatic variant calling on the matching paired samples was done with MuTect[6] and Strelka[7] and annotated using Variant Studio v2.2.1 (Illumina). A cut-off was set at alternative allele frequency >5 % and coverage >100x.

### Sequencing of plasma samples

To identify mutations in plasma, sequencing libraries from 20 ng of cfDNA were generated using the ThruPLEX DNA-seq Kit (Rubicon Genomics, Ann Arbor, Michigan, US) combined with capturing with SureSelect^XT^ Target Enrichment (Agilent) and the custom NCGC 900 panel. The libraries were sequenced and the data processed as described above. Somatic variant calling was performed on the plasma samples, verifying the mutations identified in the primary tumour as well as identifying new mutations.

In order to verify the initial findings, sequencing libraries from primary tumour, normal and plasma samples were generated using the ThunderBolts Cancer panel (Raindance Technologies, Billerica, Massachussets, US), according to the manufacturers protocol. The total input was 60 ng of cfDNA from Plasma1 and Plasma2, 180 ng from Plasma3, 50 ng genomic DNA from normal sample and 120 ng from the tumour. Sequencing was performed on MiSeq (Illumina) using TruSeq SBS v3 chemistry. Analysis was performed using the PCR amplicon workflow using BWA[2] for alignment and GATK[5] for variant calling within MiSeq Reporter (Illumina). A cut-off was set at alternative allele frequency >5 % and coverage >100x.

### References

1. Diehl F, Schmidt K, Choti MA, Romans K, Goodman S, Li M, Thornton K, Agrawal N, Sokoll L, Szabo SA *et al*: **Circulating mutant DNA to assess tumor dynamics**. *Nat Med* 2008, **14**(9):985-990.

2. Li H, Durbin R: **Fast and accurate short read alignment with Burrows–Wheeler transform**. *Bioinformatics* 2009, **25**(14):1754-1760.

3. **GATK resource bundle** [<ftp://gsapubftp-anonymous@ftp.broadinstitute.org/bundle/2.8/b37/>] (2013). Accessed 01.10.2015.

4. **Picard tools** [<http://broadinstitute.github.io/picard/>] (2014). Accessed 01.10.2015.

5. McKenna A, Hanna M, Banks E, Sivachenko A, Cibulskis K, Kernytsky A, Garimella K, Altshuler D, Gabriel S, Daly M *et al*: **The Genome Analysis Toolkit: a MapReduce framework for analyzing next-generation DNA sequencing data**. *Genome Res* 2010, **20**(9):1297-1303.

6. Cibulskis K, Lawrence MS, Carter SL, Sivachenko A, Jaffe D, Sougnez C, Gabriel S, Meyerson M, Lander ES, Getz G: **Sensitive detection of somatic point mutations in impure and heterogeneous cancer samples**. *Nat Biotech* 2013, **31**(3):213-219.

7. Saunders CT, Wong WSW, Swamy S, Becq J, Murray LJ, Cheetham RK: **Strelka: accurate somatic small-variant calling from sequenced tumor–normal sample pairs**. *Bioinformatics* 2012, **28**(14):1811-1817.
