# Supplementary figures and images for: Use of liquid biopsies to monitor disease progression in a sarcoma patient: a case report
Source: BMC Cancer. 2017 Jan 6;17:29. doi: 10.1186/s12885-016-2992-8 (PMC5219677; doi:10.1186/s12885-016-2992-8)

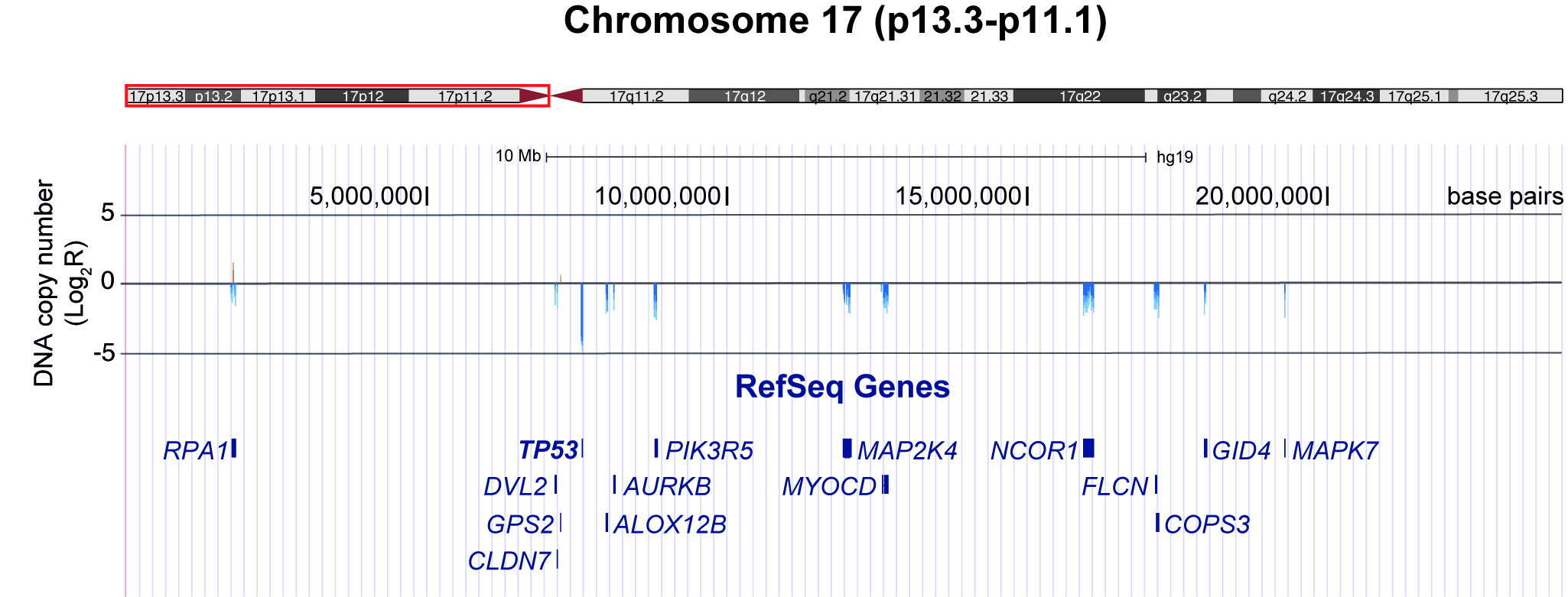

Supplement: Additional file 3 — Plot showing copy number of chromosome 17p, revealing homozygote deletion of the TP53 gene. (TIF 1032 kb) [file 12885_2016_2992_MOESM3_ESM.tif]

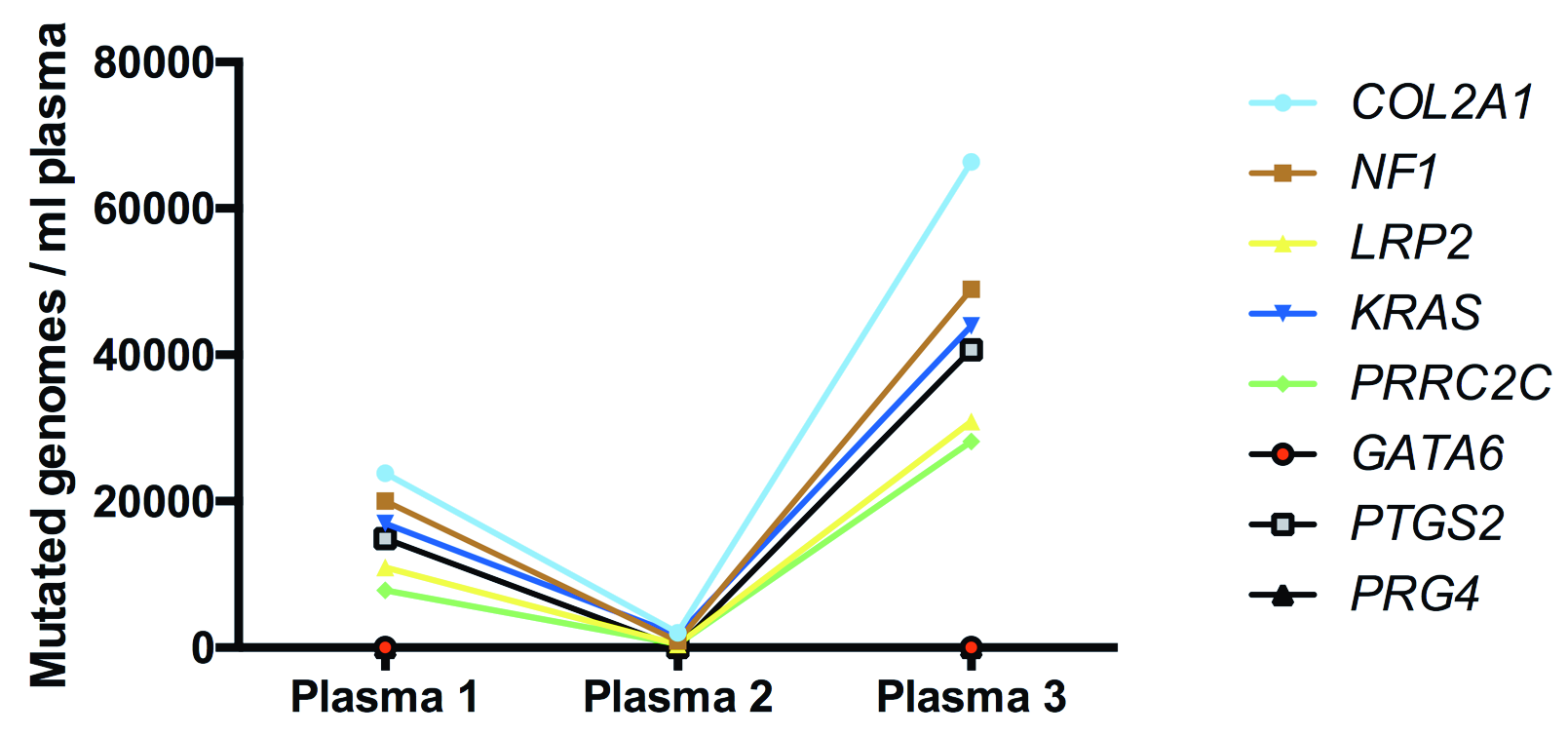

Supplement: Additional file 4 — Plot showing mutated genomes per ml of plasma, for the mutated genes, in serial plasma samples. (TIFF 283 kb) [file 12885_2016_2992_MOESM4_ESM.tiff]
